# Excessive Hemorrhage from Extraction of Third Molar

**Published:** 1901-02-15

**Authors:** D. L. Boozer

**Affiliations:** Newberry, S. C.


### EXCESSIVE HEMORRHAGE FROM EXTRACTION OF THIRD MOLAR.

BY D. L. BOOZER, JR., D.D.S., NEWBERRY, S. C.

Reprinted from *Items of Interest.*

On June 16th a young married woman who had been wearing a full upper denture for the past five years came to my office and stated that she had suffered the previous night with toothache in the region of the upper left third molar.

I removed the denture, and found the third molar protruding slightly from the gum, the entire crown decayed and gums exceedingly sore and spongy.

I advised immediate extraction. The operation was followed by a very profuse bleeding. The blood poured from both mouth and nostrils with such rapidity and in such volume that the situation was quite alarming.

The bleeding was soon checked, but the loss of blood had weakened the patient considerably. By the following day, the patient had regained her normal condition.

Upon examining the tooth I found the roots completely encircled by the alveolus, the jagged edges of which, lacerating the gum, had caused the excessive loss of blood.

To Remove Plaster.—If plaster adheres to vulcanite after vulcanizing, put the plate in warm water and add about two drachms hydrochloric acid, and in half an hour it will be clean and free from odor. When taken out, wash in water with a small portion of sodium bicarbonate.—*Dr. Genese, Ohio Dental Journal.*

